# When does broad consent expire? A temporal validity framework for biobank samples in induced pluripotent stem cell research

**DOI:** 10.21203/rs.3.rs-10412231/v1

**Published:** 2026-08-03

**Authors:** Peter A. Ikhane, Sally N. Adebamowo, Temilola Yusuf, Clement A. Adebamowo

**Affiliations:** University of Maryland School of Medicine; University of Maryland School of Medicine; Center for Bioethics and Research; University of Maryland School of Medicine

**Keywords:** broad consent, temporal validity, induced pluripotent stem cells, biobank samples, re-consent, dynamic consent, research ethics, low- and middle-income countries, Nigeria

## Abstract

**Background.:**

Induced pluripotent stem cell (iPSC) research increasingly draws on biobank samples collected years or decades earlier under broad consent for future unspecified use. We previously reported that the time elapsed between original sample collection and a proposed new use shaped whether community members and bioethicists regard the original broad consent as still sufficient, and identified this association as a novel finding, but we did not translate it into an operational standard. We address that gap in this paper.

**Methods.:**

We conducted and analyzed key informant interviews with six Community Advisory Board (CAB) members and five bioethicists, and two focus group discussions with twenty community members, from a study of the YRI HapMap community in Ibadan, Nigeria. We coded the transcripts against two *a priori* axes - duration since original consent, and novelty of the proposed technology relative to what the original consent anticipated - supplemented by inductive codes. Reporting follows the COREQ-32 guidance.

**Results.:**

Participants treated broad consent as durable but conditional rather than open-ended. Duration thresholds recurred across independent groups: renewal “at 5-year intervals,” a fixed “time limit… like 20 years,” and, at the permissive extreme, validity “to 50, 100 years.” Duration and technological novelty operated jointly rather than separately; several participants tied renewal explicitly to technology “changing,” and bioethicists distinguished elapsed time from the separate problem that the original consent language never contemplated iPSC-type uses. Anonymity of the original donors and continuity of the CAB shaped how participants resolved the two constraints, and several proposed the CAB as a surrogate re-authorizing body once individual re-contact was no longer feasible. We translate these findings into a four-state framework - presumptively valid, requires community reaffirmation, requires new authorization, and requires de novo consent - defined by elapsed-time bands crossed with a technology-novelty category.

**Conclusions.:**

Elapsed time is not, by itself, an expiration date for broad consent. It is one input, alongside technological novelty and the persistence of trust infrastructure such as CAB continuity and community reporting, into a graded governance decision. The framework gives biobanks and research ethics committees a reproducible way to decide when broad consent for a sample collection still holds, when it needs reaffirmation, and when it does not.

## Background

Induced pluripotent stem cell (iPSC) technology reprograms a stored blood or tissue sample into a self-renewing cell line that can differentiate into almost any cell type, and in principle into gametes or organ-like tissue [1]. This capability makes older biobank collections attractive research material in part because they are old: they carry years of accumulated genomic and phenotypic characterization that a newly collected sample cannot match [2, 1]. The Yoruba Resident in Ibadan (YRI) samples collected in Ibadan, Nigeria, for the International HapMap Project beginning in 2001 are one such collection [2, 3]. They were donated anonymously and stored at the Coriell Institute, where they were immortalized and the cell lines are distributed to researchers worldwide under material transfer agreements [3]. The original consent permitted future unspecified use in genetic and genomic research and stated that the samples would be turned into cell lines; iPSC reprogramming was not contemplated and could not have been because it had not been discovered, when the samples were collected [3]. Points-to-consider for deriving iPSC lines from previously collected specimens have since been articulated, but they do not resolve how elapsed time bears on the adequacy of the original consent [4].

In our previous analysis of this study, respondents gave broad support to the legitimacy of broad consent, alongside a specific and recurring reservation about the time that had elapsed between sample donation and the proposed iPSC use [3]. That study reported the association between the sufficiency of broad consent and elapsed time as “a novel finding,” and observed that, “with the proliferation of biobanks throughout the world and increasing duration of samples’ storage, elapsed time of storage may become an important consideration in whether donors or their communities need to be re-consented” [3]. We did not, however, develop the observation further, leaving open how much time matters, what triggers a need for renewal, and how duration interacts with the kind of research proposed. This gap has practical consequences. Biobanks holding legacy collections, and the research ethics committees that oversee their use need a defensible answer to the question of given a sample collected N years ago under broad consent, is that consent still sufficient for a specific new use today?

The literature offers only partial answers to this question, and those are built around consent architecture rather than time. Tiered and meta-consent models let donors specify categories of permitted future use in advance, trading flexibility for donor control [5, 6]. Dynamic consent uses ongoing electronic contact so that donors can update their preferences as research evolves [7]. The “broad but deep” model supplements a broad authorization with continuous ethical review and sustained information flow to donors [8]. Accounts of broad consent as an entrustment locate its legitimacy in an ongoing relationship of trust between donor communities and a governance structure, rather than in the specificity of the original permission [9]. Each of these models addresses uncertainty about future use in the abstract. None specifies how the passage of time itself, independent of or in combination with how novel a proposed use is, should move a sample from clearly covered to clearly not covered, even though the question of when re-consent becomes necessary has been posed in general terms [10, 11]. It is this gap that we address in this paper

Our analysis is guided by two questions. First, what temporal thresholds do stakeholders articulate, implicitly or explicitly, for the continued sufficiency of broad consent, and what reasoning underlies them? Second, how does elapsed time interact with two other features of the HapMap case - the anonymity of the original donors, which forecloses individual re-contact, and the continuity of the CAB as an institutional counterpart - to shape what stakeholders regard as an adequate response to that elapsed time? Our aim is to convert the observation that elapsed time matters into a reproducible decision framework: a small set of governance states, defined by duration and technological novelty, that a biobank or oversight body can apply to a specific proposed use of a specific legacy collection.

## Methods

### Design

We conducted qualitative analysis of data collected for the KACIY study (Knowledge, Attitude and Recommendations of Community members, Researchers, and Bioethicists on creation of iPSC from YRI HapMap samples), using the Framework method [12]. We re-coded the key informant interviews (KIIs) and focus group discussions (FGDs) against two *a priori* axes that were not previously analyzed [3]: duration since original consent, and novelty of the proposed research technology relative to what the original consent anticipated. The interviews and FGD guides were developed by the research team and attached as Supplement 1. Reporting follows the Consolidated Criteria for Reporting Qualitative Research (COREQ-32) [13], and a completed checklist accompanies the manuscript.

### Setting and context

The YRI HapMap community is in Ibadan, Oyo State, Nigeria. A CAB was constituted for the HapMap project in 2001 and has continued to function since, with some membership turnover, receiving and disseminating reports on the users and uses of the YRI samples [3]. We conducted the KACIY study between November 2023 and May 2024.

### Participants and sampling

Participants were three stakeholder groups, sampled purposively. We enrolled six of the eight members of the CAB constituted for the HapMap project for KIIs; one member was unavailable and one was deceased. We recruited twenty members of the community where the YRI samples were collected for two FGDs of ten participants each, balanced by sex, age, religion, and socio-economic status. We enrolled five bioethicists for KIIs, four of whom had been directly or indirectly involved in enrolling participants at the YRI site and one of whom was the principal investigator of the repository where the samples are banked. Demographic characteristics of the participants are reported in the marker manuscript [3].

### Data collection

The KIIs with CAB members and bioethicists lasted 60 to 90 minutes, and each FGD lasted approximately 90 minutes. The interview and focus group guides, developed from the literature on research ethics and on the ethics of iPSC research, covered consent scope, permissible future use, and researchers’ obligations to donor communities over time, among other topics, and were piloted with three individuals outside the study community. Because CAB and community members may have had limited familiarity with iPSC, we prepared an information sheet from publicly available sources and shared it with them before the study began. P.A.I. led all interviews and focus groups; T.Y. was the note-taker for the CAB KIIs and the FGDs, and C.A.A. was the note-taker for the bioethicist KIIs and attended the CAB KIIs virtually without participating. All sessions were audio-recorded and transcribed, and transcripts were returned to participants for comment.

### Data management and analysis

We transcribed the recordings using a combination of a speech-to-text transcriber (Otter.ai) and manual methods and reviewed the transcripts for accuracy. For this analysis we applied the Framework method to the transcripts and the analytic memos, structuring an initial coding matrix around two *a priori* axes - elapsed time since original consent, and technological novelty of the proposed use - and retaining inductive codes where participant language exceeded the frame. We charted coded segments describing renewal, time limits, “coming back,” or comparable temporal language into a matrix by stakeholder group and elapsed-time reference. One analyst coded the material and a second reviewed the coding, and we resolved discrepancies by returning to the transcript. Because the focus group format makes individual attribution unreliable, we report the range and patterning of views across groups rather than counts of individuals. We identify participants by group and code, as CAB, P3 to P8; FGD, R1 to R20; and BE, P1 to P5. Analysis used a combination of manual coding and NVivo.

### Reflexivity

Some members of the team that conducted and analyzed this study took part in the original HapMap community engagement, and who therefore have a long-standing relationship with the CAB and the community. This relationship plausibly increased participants’ candor about researchers’ obligations over time, but it may also have made participants reluctant to state directly that the team’s own prior consent was no longer adequate. We therefore treat statements that broad consent should nonetheless be renewed, offered despite this relationship, as a conservative signal of participants’ underlying views, and we anchor every claim to verbatim language.

## Results

Thirty-one participants contributed the material analyzed here: six CAB members, twenty community members across two FGDs, and five bioethicists.

### Broad consent is treated as durable, not permanent

Participants did not treat the original HapMap consent as void by default. Several CAB members described it as still carrying weight, in part because it was framed with the long term in mind. One member grounded its durability in practicality, noting that most original donors could no longer be found:

*“What we have given before is still very, very important. There is no need for you to still come back and be looking for another permission for what we have done before… You cannot see the total number of the people who gave samples. The consent you have taken before is still important. And I want you to continue on building on that consent.”* (CAB, P7)

Another placed an explicit and very long horizon on that durability, tying it to the long-term intent behind the original engagement:

*“It is long-term thinking that is helping you now. That is why we can discuss what we are discussing today… This consent will take you to 50, 100 years.”* (CAB, P8)

Durability was consistently qualified. Even participants who defended the continuing force of the original consent paired that defense with an expectation that researchers would return to the community from time to time; the same CAB member who endorsed building on the earlier consent also held that “*it is good they come back to renew the consent now and again*” (CAB, P4).

Bioethicists framed the same conditionality in institutional terms. One, who had read the HapMap consent form line by line, distinguished the general legitimacy of broad consent from its fit for a use as far removed as iPSC research:

*“The HapMap consent cannot, unless there’s a particular body that approves it for iPSCs, on its face, it cannot be used for iPSCs… it mentions cells… diagnosing, treating diseases… it mentions cell lines, but it doesn’t talk about creating tissues or creating organs or other potential therapeutic uses of those cell lines.”* (BE, P1)

### Duration thresholds, from open-ended trust to fixed calendar limits

Distinct duration thresholds recurred across groups. The shortest and most specific was a five-year renewal cycle, proposed in the FGDs and tied directly to technological change:

*“I support the type of consent where researchers renew the consent taken at 5-year intervals as technology is changing, because if they use the present consent for today’s technology, they will need to come back when the technology changes.”* (FGD, R1)

At the group level, one focus group summarized the same position and attached a reporting obligation to it:

*“Researchers should renew the consent taken at 5-year intervals as technology is changing… Regular updates should be provided to the community where the samples were donated”* (FGD 1).

A second, longer threshold set an outer boundary on validity rather than a renewal cycle:

*“For me, I think there should be a time limit for the use of broad consent, say like 20 years.”* (FGD, R4)

A CAB member independently raised a comparable interval, and used it to press the question of whether the community had been kept informed over that period:

*“…for all that we know, the question may arise; after HapMap, have they been getting yearly information as to what has gone on with their samples? Or you guys are just coming back after 20 years, 15, 20 years to tap on them again.”* (CAB, P5)

The same member who endorsed periodic renewal also tied the length of the interval to who should be authorized to act on the community’s behalf:

*“…it is good they come back to renew the consent now and again… this research about HapMap that happened a long time ago… members of CAB can decide for the people. But if it’s another length of time that is not too long, the people can be contacted.”* (CAB, P4)

This is the most explicit duration-dependent decision rule in the data: short intervals should trigger direct re-contact with donors or the community, and long intervals should route re-authorization through the CAB as a standing surrogate. The horizon of “50, 100 years” cited above (CAB, P8) shows that the numeric thresholds participants proposed did not converge on a single value. What was convergent was the underlying structure - duration alone did not settle the question, but duration combined with an account of who remained available to reauthorize did.

One FGD participant raised a different kind of elapsed-time concern, tied to biological safety rather than consent validity, which we distinguish from the constructs above:

*“…they should test it very well and wait for some time like 10 years to test it before they start using it on human beings.”* (FGD, R1)

This is a claim about the maturation of a technology’s safety evidence, not about the sufficiency of a prior consent, and we treat it as conceptually separate in the framework below.

### Technological novelty compounds elapsed time

Participants and bioethicists frequently combined a duration reference with a novelty reference in the same statement, treating the two as jointly rather than independently determinative. The clearest instance is the “technology is changing” clause attached to the five-year renewal proposals above. A community member made the same point about novelty through an extended metaphor, without attaching a number of years:

*“It [the previous broad consent] is not enough because the future is very wide and far. It’s like a journey, and when you reach a spot, you stop, and you look, and it doesn’t look like where you are coming from.”* (FGD, R1)

This construction ties reassessment to milestones in scientific development rather than to calendar time, in contrast to the fixed five- and twenty-year thresholds.

Bioethicists located the same tension in the specific language of the original consent form. One described elapsed time and the nature of iPSC research as two separate difficulties affecting the case for reuse: the interval between the original project and the new studies, and the character of iPSC work as a technique that did not exist at collection. Another raised the point from the standpoint of technological non-existence rather than mere unfamiliarity:

*“I do have concerns about reprogramming iPSCs from samples that were collected before the technology existed, because we don’t have a way to go back to the specific individuals that donated the samples to ask them. I think the best we can do is to try to give the same communities a chance to tell us what they think about the technology now.”* (BE, P5)

A third reported an emerging professional consensus that certain transformative applications should require specific consent regardless of the breadth of the original authorization:

*“…there seems to be an emerging view that… without getting specific consent, we wouldn’t want to allow this kind of research for anything that would involve creation of gametes or like brain or transplantation.”* (BE, P3)

This mirrors professional guidance for stem cell research, which subjects such applications to specialized oversight and review [14].

One bioethicist proposed tiered consent as a structural response to novelty compounding over long periods, while acknowledging its limits:

*“…tiered gives us the flexibility to do a number of things… including things that we never anticipated… going back to tiered, and thinking about what the tiers are, might be a useful way of addressing things like iPSC 20, 30 years down the road, who knows what will come down the pike in terms of science.”* (BE, P2)

### Anonymity and CAB continuity moderate the effect of time

Because the original HapMap consent did not retain identifying information sufficient for routine re-contact, individual re-consent of the kind used in some other legacy cohorts was not available to this study population. One bioethicist described a comparator case in which re-contact had been possible, and the decision that followed:

*“…with those samples [from the Utah community], they did have the names of the people, because they had kept the individual names. And so, the decision was made to go back and re-consent them, even though there was debate about that… in the end, we decided, let’s go back and re-consent them.”* (BE, P3)

She then drew the contrast with the YRI case directly:

*“The difference being that in that case, we were able to go back to each individual and get the reconsent, whereas here we’re not… we do have the ability to at least go back to the community advisory group and general members of the community… So, the question is, is that sufficient or not?”* (BE, P3)

This frames the CAB and the community as a second-best substitute for individual re-consent, adopted because anonymity forecloses the first-best option, rather than as an equally strong alternative in its own right. A different bioethicist noted that the community itself may change over a long interval in ways that complicate even institutional re-authorization:

*“…those communities have probably changed… might have politically mutated… or have changed the surrounding, the authority, the hierarchy. I think if you wanted to continue… the original four HapMap communities.”* (BE, P1)

Participants on the Nigerian side treated CAB continuity as the condition that made institutional re-authorization credible in the first place. One CAB member tied the acceptability of relying on institutional consent, rather than tracking down individual donors, to the persistence of communication with the community:

*“If communication has not been optimal… there you will have a bit of an uphill. But if you’ve been carrying them along… if these are the developments, and they see and know how their samples are being used… then I don’t think that they will give you any problems in giving you either specific or broad approval.”* (CAB, P5)

Another member’s account of the community’s relationship to elapsed time shows why this continuity matters: without it, the passage of time produces not principled objection but simple forgetting.

*“We have forgotten about it since it was many years back. It’s only now when you people come up with this project that we remember.”* (CAB, P7)

Renewal, on this reading, functions as a package rather than a single act. Participants tied it consistently to reporting and reciprocity, not to permission alone; the focus group that proposed the five-year cycle attached “regular updates” to it (FGD 1), and the marker study similarly found that reports “*give the community an opportunity to assess the work of the research team*” [3].

### A temporal validity framework

The findings do not converge on a single duration threshold, but they do converge on the structure of the decision that duration feeds into. We propose four governance states, defined jointly by elapsed time since original consent and the novelty of the proposed research relative to what that consent anticipated (Table 1).

The framework treats duration and novelty as interacting rather than independent inputs, consistent with participants who paired a numeric interval with a technology-change condition (FGD, R1). A short interval does not rescue a transformative use from requiring de novo consent, and a long interval does not by itself disqualify an incremental use that has been sustained by periodic community reaffirmation. The bands in Table 1 are drawn from the numeric thresholds participants proposed - five, twenty, and, at the permissive extreme, fifty to one hundred years - and are offered as a starting point for calibration rather than as fixed values; a biobank governance body could set its own bands in consultation with the donor community it serves.

#### Worked example

Applied to the case that prompted this study - reprogramming YRI HapMap samples, collected beginning in 2001, into iPSCs for disease-modeling research in 2024 - the elapsed interval of more than two decades falls at or beyond the outer threshold most participants proposed, and the use is transformative relative to a consent form that addressed disease-association genetics and cell lines, not tissue or organ generation. The framework places this case in the “requires de novo consent” state: continued CAB engagement and community reporting remain necessary to sustain trust, but they are not, on the framework’s own logic, a substitute for a new, iPSC-specific consent process.

## Discussion

Our findings answer the question our previous study raised but did not resolve. Participants did not experience elapsed time as a single clock running out, but as one of two interacting inputs - duration and technological novelty - that jointly determine whether an old broad consent still covers a new use. This distinguishes the temporal validity framework from existing consent architectures, each of which addresses one input without the other (Table 2).

The framework’s practical value is that it converts a qualitative sense that too much time has passed into a decision a repository or research ethics committee can apply consistently. Applied prospectively, it gives a biobank a basis for deciding, before a proposed use reaches review, whether existing broad consent is likely to be sufficient, whether community reaffirmation should be sought, or whether a new consent process is required. Applied to material transfer agreements, it gives a repository a basis for flagging or declining requests that fall in the “requires new authorization” or “requires de novo consent” states, regardless of whether the language of the original consent is technically broad enough to cover them. Applied to community engagement, it gives a CAB a concrete standard against which to judge whether the reporting and reaffirmation its community has received are sufficient to sustain institutional re-authorization as a credible substitute for individual re-consent, which is the condition CAB members themselves identified as decisive (CAB, P5).

The framework also clarifies what renewal means in this population. Participants did not describe renewal as a single signature event. They tied it to reporting, to evidence that the research was benefiting the community, and to sustained communication that prevents the community from simply forgetting the original study (CAB, P7). A biobank that reaffirms consent without also reporting back is unlikely to satisfy the condition participants described. This is consistent with our previous finding that re-engagement through community leaders, CABs, and community representatives was the most preferred mechanism, and with the wider literature on community engagement in African genomics [3, 9, 16].

We use “expire” as shorthand for a graded governance judgment, not as a claim that broad consent becomes void at a fixed date. No participant described consent as suddenly invalid. They described a widening gap between what was originally authorized and what is now proposed, met by an escalating set of institutional responses. Framing the question this way avoids two failure modes: treating any elapsed time as fatal to broad consent, which would make legacy collections unusable however faithfully a biobank has maintained community trust, and treating elapsed time as irrelevant, which our previous study rules out [3].

### Limitations

We analyzed a modest, non-representative sample from a single community. The numeric thresholds participants proposed - five, twenty, and fifty to one hundred years - should be read as illustrative anchors rather than validated cut points; different communities, technologies, and original consent language could plausibly produce different bands. Participants described hypothetical preferences about renewal rather than responding to an actual renewal process, so we cannot observe whether stated thresholds would hold under a real re-authorization request. Elapsed time in this dataset is confounded with CAB membership turnover and with the specific history of the HapMap-to-iPSC transition, so the duration bands should be recalibrated, not assumed to transfer unchanged, for collections with different histories. Finally, the framework has not yet been reviewed by the CAB or the donor community and is presented as a proposal for such review.

## Conclusions

Elapsed time shapes whether stakeholders regard broad consent for legacy biobank samples as still sufficient, but it does so jointly with the novelty of the proposed research and the persistence of trust infrastructure such as CAB continuity and community reporting, not as an independent clock. The four-state temporal validity framework proposed here - presumptively valid, requires community reaffirmation, requires new authorization, and requires de novo consent - gives biobanks, repositories, and research ethics committees a reproducible way to apply that judgment to a specific proposed use of a specific legacy collection, and gives donor communities a concrete standard for what sustained engagement over time should look like.

## Supplementary Material

This is a list of supplementary files associated with this preprint. Click to download.

- Supp1.FGDandKIIGuideForKACIYProject.docx

## Figures and Tables

**Table 1:** Temporal validity framework for legacy biobank samples proposed for novel research use

Governance state	Elapsed-time band	Technology novelty	Required action	Illustrative evidence
Presumptively valid	Short (within an initial renewal cycle, e.g. ≤ 5 years)	Incremental - consistent with original consent language	Proceed under original broad consent, with standard research ethics committee review	“*researchers renew the consent taken at 5-year intervals as technology is changing*” (FGD, R1)
Requires community reaffirmation	Intermediate (beyond the renewal cycle but within an outer limit, e.g. 5–20 years)	Incremental to moderately novel	Seek CAB or community-level reaffirmation with public reporting; attempt individual re-contact where feasible	“*if it’s another length of time that is not too long, the people can be contacted*” (CAB, P4)
Requires new authorization	Long (beyond the outer limit proposed by participants, e.g. > 20 years) or CAB/community discontinuity	Incremental to moderately novel	Obtain institutional re-authorization through the CAB or successor body as surrogate for donors who cannot be re-contacted; disclose that individual re-consent is infeasible	“*members of CAB can decide for the people” when the interval* *“happened a long time ago*” (CAB, P4)
Requires de novo consent	Any interval	Transformative - outside any use category the original consent could plausibly have anticipated (e.g. reprogramming into gametes or transplantable tissue)	Conduct a new, purpose-specific consent process; broad consent, however recently reaffirmed, is not sufficient on its own	“*on its face, it cannot be used for iPSCs*” (BE, P1); “*without getting specific consent, we wouldn’t want to allow… creation of gametes or like brain or transplantation*” (BE, P3)

**Table 2:** Comparison with existing broad-consent governance frameworks

Framework	What it establishes	What it leaves unspecified	What temporal validity adds
Broad consent, single unconditional authorization [15]	Permission for future unspecified use in general terms	Any limit on how much time or how much novelty may accrue before the authorization stops applying	Explicit duration bands and a novelty trigger
Tiered/meta-consent [5, 11]	Donor-specified categories of permitted future use	How a category-based system should treat a category, such as iPSC reprogramming, that did not exist when the categories were defined	An explicit “requires de novo consent” state for transformative novelty, regardless of category language
Dynamic consent [7]	Ongoing donor-level control through electronic re-contact	Feasibility when donors are anonymized and cannot be re-contacted, as in most legacy collections	A CAB-mediated substitute state for populations where individual dynamic consent is not possible
Broad but deep, with continuous review [8]	Sustained ethical review and information flow to donors	A duration- or novelty-based rule for when review should escalate to reaffirmation or new consent	Concrete elapsed-time bands and a novelty criterion to structure the escalation
Entrustment [9]	Legitimacy grounded in an ongoing relationship of trust rather than a specific permission	A way to test empirically when that relationship has, or has not, been sustained	Community reporting and CAB continuity as observable proxies for whether the entrustment still holds

## Data Availability

The qualitative datasets analyzed in this secondary analysis are not publicly available because of the identifiability of a small community-based sample, but coded excerpts are available from the corresponding author on reasonable request.
